# Designing and Evaluation of an Artistic Experience for the Development of Empathic Capacity: “Stepping into Others’ Shoes”

**DOI:** 10.3390/brainsci12111565

**Published:** 2022-11-17

**Authors:** Rut Martínez-López de Castro, Myriam Alvariñas-Villaverde, Margarita Pino-Juste, Sara Domínguez-Lloria

**Affiliations:** 1 Research Group on Education, Physical Activity and Health (GIES10), Galicia Sur Research Institute (IIS Galicia Sur), SERGAS-UVIGO, 36312 Vigo, Spain; 2 Department of Special Didactics, Faculty of Education and Sport Sciences, University of Vigo, 36005 Pontevedra, Spain; 3 Department of Didactics, School Organization and Research Methods, Faculty of Education and Sport Sciences, University of Vigo, 36005 Pontevedra, Spain

**Keywords:** identity, artistic project, empathic pedagogical relationship, didactic interaction, art

## Abstract

This article evaluates an educational experience that uses art to develop empathy. The artistic educational project is called “stepping into others’ shoes” and is carried out with 71 students enrolled in the Early Childhood Education and Primary Education degree programs of the University of Vigo. The main objective is to analyze the students’ experiences in processes of empathic pedagogical relationships that allow empowerment dynamics of oneself and others. An ethnographic approach is used to evaluate the experience through a group case study design with different qualitative instruments: analysis of artistic production, field diary and life stories. The main results indicate that when art is used for the construction of an empathic identity, the participants of these creative dynamics find it difficult to express their feelings and emotions. However, these processes favor social relationships and mutual recognition, as well as self-knowledge. It facilitates the creation of alternative meeting spaces and the promotion of creativity. Based on these results and their discussion, lines of action are suggested which should allow using art as a resource to develop empathy and promote increased motivation in the classroom.

## 1. Introduction

University education in Spain is facing new social and political changes that imply new paradigms beyond a technocratic organization. Many times, teachers must fight against a conservative educational system that is oriented towards maintaining the status quo. Faced with this reality, the research intends to open possibilities from the artistic field that pay attention to empathy in the pedagogical relationship. That is, this study presents a proposal focuses focused on the relational and not on the individual aspect. This study examines the association of creativity developed by the arts with students’ empathy through their participation in a program developed in an academic setting. These results are important for researchers given the need to create scientific evidence on the arts in the integral development of the human being.

The educational change, according to Fullan [1], cannot be individual, it must occur in broader social terms a community context, based on a shared moral purpose. To this end, “the artistic education within during the university education, the artistic education must involve not only a content planning that facilitates learning of a certain information but should also enable lifelong ethical teaching that educates in human values” [2] p. 53. That is, “it should be based on promoting sensitive teaching that creates moments of exchange of subjectivities and knowledge which affect the sense of self” [3].

### 1.1. The Empathic Pedagogical Relationship from the Perspective of Artistic Research

Rethinking the pedagogical relationship in the classroom involves exploring new artistic narratives from an empathic perspective to the construction of subjectivity and personal identity. Irwin [4] p. 108 points out that arts are a way of “investigating the world to improve its knowledge” and creating new and/or relevant meanings.

An artistic education based on human relationships should create opportunities for the exchange of ideas and transformation of students’ identities through shared aesthetic experiences. As defined by Bourriaud [5] p. 142, this is a proposal of relational art that would take, “as a theoretical and practical starting point, the range of human relationships and their social context, rather than an autonomous and restricted space”.

The experiential and multisensory nature of arts brings us closer to the human condition, and to the interactions that occur among people: “The function of the arts throughout the cultural history of humanity has been and continues to be the “construction of reality”. The advent of postmodernity has not fundamentally modified its function. Artists construct representations of the real world or of imaginary worlds that provide human beings with incentive to create a different reality for themselves” [6] p. 124.

The practices of visual, performative, or narrative inquiry favour the construction of meanings and experiences. They reveal unexplored aspects, providing other ways of constructing personal and collective narratives. Thus, identities become the focus of attention of this artistic pedagogical project aimed at making students acquire self-awareness and increase sensitivity toward others. 

The pedagogical approach through art based on empathy has the potential to train people to be ethical, allowing them to bring them closer, with the responsibility of sharing ways of seeing and being. Walia’s study [7] where definitions of creativity are reviewed points out that in general the terms creativity and creation have been merged in the understanding of the construct, and no attention has been paid to the dynamic process of creativity that may or may not lead to creation. Walia (2019) [7] or Glăveanu [8] propose a dynamic definition considering several elements involved in creative acts. However, identifying the exact factors of creativity remains difficult [9]. Empathy can be assumed as a transient and immediate emotional state, which is explained through the mirror neuron theory [10]. On numerous occasions it is assumed as a trait or disposition to act in a predictable way in similar social situations, which would be more linked to personality and would be assumed as a construction achieved through social training and learning [11]. If we talk about the dimensions of empathy, we find two dimensions of cognitive type one of them based on the ability to understand the emotions of the characters in novels or movies and the other and perspective taking allows developing the ability to understand the positions and arguments of the other. The other two dimensions would be emotional: empathic concern, which is defined as the ability to feel what the other feels (that is, the ability to “put oneself in the other’s shoes”) and which is the one that occupies the article; and personal empathic stress, which would measure the capacity for personal discomfort generated in the face of adverse situations of others [12].

The pedagogical approach using art based on empathy has the potential to somewhat train people to be ethical, allowing to bring them together, with the responsibility of sharing ways of seeing and being. Walia’s study [13] where definitions of creativity are reviewed points out that in general the terms creativity and creation have been merged in the understanding of the construct, and no attention has been paid to the dynamic process of creativity that may or may not lead to creation. Walia or Glăveanu [7] propose a dynamic definition considering various elements involved in creative acts. However, identifying the exact factors of creativity remains difficult [14]. Indeed, in recent years, social and environmental influences on creativity have been subjected to examination [15] given the central role of creativity in different cognitive processes involved for originality and potential effectiveness [16]. In this study we have started from this dynamic definition of creativity where different ordinary cognitive processes common to all people influence, which are refined through experience and effort. Therefore, we all have a creative potential that will develop in favorable environments. 

The arts have the capacity to create meeting points between the subjects. One could speak of “the arts as a relational pedagogy” [4] p. 111, which emphasizes the bond and the mental and affective identification with the feelings of others. In other words, it may be said that the body, literary or visual art are processes of esthetic and pedagogical exploration that raise questions and create new perspectives where different “selves” are projected to build an empathic “we”. 

It has been common for the arts to help people learn about and care about situations and people other than themselves [17]. However, research demonstrating the benefits of the arts on empathic ability is still in its infancy. Kim, Yang, and Kang [16] analyzed the effects of students’ empathic ability on group creativity and concluded that groups with high empathic ability show more frequent interaction in tracking, planning, and divergent thinking. Furthermore, Kou, Konrath, and Goldstein [15] conclude that artistic engagement is associated with prosocial behaviors. Stamatopoulou [17] argues that an affective perception results in a feeling of proximity/connectedness that are characterized by spatiotemporal coordination and proximity of the interactive self/other/object and, therefore, there might be something special between art and negative emotions in relation to empathy. In the same vein, Lubicz-Nawrocka [18] suggests that involving students in the creation and design of their curriculum help them develop skills such as confidence, empathy, or resilience.

The artistic educational proposal from which we start recognizes the importance of pedagogical sensitivity [19], focused on the affective component as a value necessary for learning. Empathy is one of the values that emerge from this interaction between the group of students and the teacher, where everyone shares subjective experiences and knowledge from the recognition of the other. 

In the pedagogical relationship there cannot be a teacher-student differential relationship, but a team where both are responsible. The teacher is the promoter of the dynamics of change [1] and the provocateur of democratic participation spaces. In this regard, Hernández [3] stated that: “We should pay attention to the pedagogical relationship at the university if we want to break the naturalized positions that present the learning process as repetition, the teacher as custodian and transmitter of information and knowledge and students as subordinate players” (p. 12).

In other words, the pedagogical relationship from the perspective of artistic education should allow creating spaces of affective and cognitive meeting, where a mutual empowerment of the participants takes place. It is a democratic teaching process, which prioritizes what is known, felt, or expected. Thus, the arts can enable empathic collaboration encounters to build a being in relation to others and their environment. This means recognizing the other person as someone who has entered our lives and from whom we must assume their influence and be willing to learn. The objective of this study is to analyze the experiences of students in empathic pedagogical relationship processes that allow for dynamics of empowerment of self and others.

### 1.2. Description of the Project “Stepping into Others’ Shoes”. Artistic and Performative Actions

The objectives of the project were: (a) to understand artistic education as a means of knowledge to develop empathic capacity, (b) to enable spaces for artistic creation from different expressive languages, (c) to generate a shared reflection on the value of empathy in education, and (d) to understand research based on arts as a means of knowledge and to favor personal relationships and recognition of the other.

The methodology used was active and collaborative to favor interaction, manipulation, experimentation, and internalization of curricular contents of art education. This methodology aims to connect knowledge, relate, and value, is critical with the environment and makes proposals for personal and social transformation [20,21]. 

The experience aims to train future teachers of early childhood and primary education, so it has been intended to apply the principles of “learn by doing-research by reflecting”. This methodology encourages creative and autonomous work and sensitivity to solve problems of their own reality. The aim is to train future teachers in the management, control, and expression of their students’ emotions, facilitate social relations and teamwork, help them to place themselves in the intentional and emotional perspective of others, or to learn collaboratively and perform shared tasks.

The activities developed are briefly described below, with the aim of allowing the reader to understand the results analyzed.

The first educational activity was called “It’s me with another” and started with a performative action with the aim of achieving a meeting between students. The aim was to incorporate performance as a collective esthetic experience that would allow understanding the concept of empathy in the context of action, exploring unpredictable connections, seeking answers, and embodying the presence of the other in the self.

The performative action consisted of choosing the shoes of another classmate and walking in them for a while, assuming their feelings and emotions. 

After completing the walk, the students expressed their feelings with words written on stickers. We point out some of them: Comfort, indifference, tight, weird, joy, calm, safe, stability, happiness, space, very close to the ground, warm, walking slowly, warmth, different, novelty, stability, freedom, etc.

The second activity was a proposal for dialog with the owner of the shoes, to share those empathy and antipathy experiences. The dialog served to create a space for mutual recognition, where both perceive and understand the wishes, feelings, or ways of thinking of their classmate. During this action, words and phrases were recorded, sharing empathy and antipathy experiences on topics about partner and family relationships.

During the third activity, a life story was described with the classmate- with whom the previous activity was carried out. To this end, a semi-structured interview was designed, with questions about their tastes, wishes, significant life experiences or other issues that students considered relevant. These data were recorded in audio format and then transcribed for analysis. With this information, the students created their artistic productions.

The last activity consisted of an artistic creation based on life stories. The work was about the person with whom they had exchanged shoes, discussed, and developed the life story. The productions were raised based on aesthetic and technical freedom, considering the diversity of a student’s life paths. The only requirement was to include some shoes or a related accessory. Each student initiated the creative process with sketches and writings about the relevant aspects and characteristics of their classmate’s biography. The production was supported with photographs, drawings, and artistic installations.

The artistic creation was the closing of the activity. The objective of the latter was for each participant in the experience to create his or her own work based on a personal sketch that expressed as clearly as possible the idea that he or she intended to represent. The task was for each person to record and capture in his or her visual work those dimensions of the partner in dialogue with himself or herself.

## 2. Materials and Methods

An ethnographic approach was used to evaluate the experience through a group case study design and arts-based research. The study was developed taking a holistic inquiry approach integrating different fields of artistic knowledge.

According to Stake [22] p. 11, “case study is the study of the particularity and complexity of a single case, coming to understand its activity within important circumstances”. In addition, they are especially valuable because they allow the study of causality and, as they develop, “a more mature theory emerges, which progressively takes shape (though not necessarily perfectly) until the case concludes” [22] p. 5. Arts-based research is an umbrella concept that allows for a wide variety of procedures, approaches, techniques, and outcomes [23]. Arts-based research proposes an approach and openness from scientific research to artistic creation to use its forms, knowledge, and know-how. Arts-based research always starts from the association with artistic activity and where certain aesthetic qualities, or design elements permeate the research process [24]. 

One of the most important characteristics of this type of research is that it employs all artistic representations and languages using artistic outcomes as how the research is made possible. Perhaps a fundamental characteristic of this research methodology is not so much to explain educational or social events as to propose or suggest new ways of seeing or understanding them. 

This study is developed from a holistic inquiry approach that integrates different fields of artistic knowledge.

### 2.1. Participants

The project was developed with a purposive sample of 71 students of the third year of the Degree of Early Childhood Education (42) and Primary Education (29) of the University of Vigo (Spain) with an average age of 20.7 years. The selected students are enrolled in the subject “artistic languages”. This subject is compulsory for future teachers of primary and early childhood education. The group is very homogeneous in terms of social and cultural characteristics. It is common in Spanish universities for groups to be very homogeneous since the student body is very similar and there is not much inter-territorial mobility. In other words, students belong to similar age groups, culture, or religion, speak at least one common language, share a series of common beliefs and there are no significant social differences since all students have access to a quality public university. In addition, being a classroom activity, the students participated in it with a high level of motivation. The described activity was scored and evaluated.

### 2.2. Procedure 

One of the fundamental aspects of the rigor in qualitative research is its adaptation to the evidence. The credibility of qualitative research through case studies is a concern in the context of educational inquiry. We therefore seek to avoid the possible subjectivity of the researcher or the impossibility of generalizing the results obtained in this work. The process of data collection was detailed, along with the sources of information and interpretation thereof through the description of contextualization, saturation of information, negotiation with the persons involved and technique triangulation [21,22].

As for the ethical aspects of the research study, it was conducted in accordance with the Code of Good Scientific Practice developed by the Spanish National Research Council (CSIC Ethics Committee) and complies with the approval of the ethics committee of the University of Vigo.

### 2.3. Instruments

The instruments that have allowed us to evaluate the experience have been the analysis of the life stories, the artistic production, and the field diary. During four two-hour sessions, the students were offered different activities already described: performative activity, dialogue, life story with the partner and artistic creation based on the life stories. All the material was collected by the teacher on the online teaching platform that is usually used, which allowed us to analyze it and to be able to access it in case of any doubt in the interpretation.

A student’s biographical stories were addressed as a shared process between interviewer and interviewee, with the aim of incorporating the look, the reflection, and the search for new meanings about what they lived. The story became a space for identity reconstruction where the person who writes, while narrating the experiences about the other, adds something of oneself, because they identify with the other in the story.

The stories that emerged did not start from the same baseline. Each student explored and shared different aspects of their classmate’s life. The objective was to enable the opening of spaces for self-expression, regardless of whether this is already a reality or still an aspiration. 

For the content analysis of the artistic production, it was considered that the artistic image is a very relevant document, since it conveys a great range of communicative messages, and provides the opportunity of verbally reconstructing and representing a discourse. The artistic production plays a fundamental role in the creation, representation, and projection of ideas. The motivation of the students could be measured through the field diary made by the participants themselves, a post-test of the experience and through participant observation in the students’ work groups.

The field diary was another of the instruments used to record critical incidents, perceived as relevant. It refers to the record of everything that has to do with the objectives of the research, such as the empathic relationship, mutual recognition, understanding of, and respect for others, self-knowledge, creativity, flexibility, originality, etc.

### 2.4. Data Analysis

The data gathered was analyzed in parallel with the fieldwork process itself, as is usual in qualitative studies. The purpose was to classify the different parts according to previously established categories, so that these categories may be identified systematically and objectively within a message. Consequently, the process falls within the scope of content analysis techniques whose phases were: Reading and comprehension of texts, images or drawings, definition of categories, their analysis—choice and interpretation—and synthesis [24,25].

This is how the dimensions of analysis emerged, as part of a complex induction process as the research unfolded: Analysis of the didactic experience, characteristics of the empathic pedagogical relationship, and creativity.

## 3. Results

To interpret the results of the process, we analyzed the data collected through the records in the field diary, the life stories and the artistic productions supported by photographs, drawings, and performance that allowed us to define the categories of analysis (Table 1). 

### 3.1. Esthetic Inquiry

The experience allowed us to learn another way of assimilating new knowledge in artistic education, focused on aesthetic and pedagogical inquiry. In fact, the activities served to go beyond the development of exclusively manual skills and allowed emphasizing the essence of contemporary art that overcomes the compartmentalization of the different art forms: Painting, drawing, photography, poetry, and performance. 

The students expressed their satisfaction by referring to this diversity and interrelation between the different art forms and what was shared with their classmates in the following way: “My assessment of the project is positive, because it allowed me to see artistic education in a more global way, from the writing, performance, and visual perspective. In addition, I would like to stress the dynamics of creative research and what I have learned in the exchange with my classmate” (ANMA)

Inquiry was also important to break out of the routine and monotonous classes, and in contrast, to conceive artistic education as a way of expression, enjoyment, creativity, and empathy. In this project, students were shown a new approach to the teaching of arts that favors an exploration of lived experiences. We believe that each image, story, or action represented ways of understanding the world and oneself, learning processes, and shared experiential and creative inquiry.

**Figure 1:**
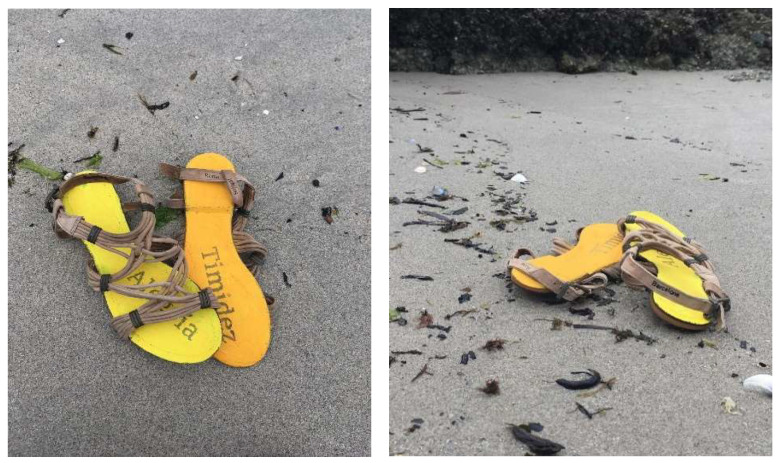
Intertwined. Photography (SABE).

The students perceived this approach to artistic inquiry as a way of pedagogical reflection to make sense of the experiences they live with their classmates. An example is shown in the story supported by photographs (Figure 1): “My work is composed of two sandals that my classmate has given me (...) Once I saw them, I told her that their shape could give us a lot more information and meaning to my work, since their intertwined structure could be related to the way of thinking of my interviewee, since she considers that many concepts, such as education and all current problems, are linked...” (SABE)

SABE’s story and images are the result of a pedagogical action that explores ways of being and of knowing the others. Artistic inquiry constantly leads us to reflect on our relationship with knowledge.

### 3.2. Empathic Pedagogical Relationship

#### 3.2.1. Mutual Recognition and Self-Knowledge

The introduced experience allowed students to know each other more, discover characteristics of themselves, and how they were perceived by others. The entire student body used artistic knowledge as a tool of knowing each other through their classmate´s stories. At the same time, they were able to discover their own strengths or weaknesses seen by others.

The students also understood this and noted that starting from the conversation with my colleague, I discovered many things about her way of being, her way of thinking and her personality (BOZA).

The students also understood this and noted that “starting from the conversation with my colleague, I discovered many things about her way of being, her way of thinking and her personality” (BOZA).

In addition, they were able to discover characteristics that had gone unnoticed in their classmates: “While talking to D, I discovered that he is a very active guy, who likes to have fun in very different ways” (ALPI).

During the conversations, an attempt was made to create a space for recognition, where both perceive and understand the wishes, feelings, or ways of thinking of the other. In fact, during the assessment of the process, the participants expressed their gratitude forgetting to better know their classmates: “First of all, thanks for carrying out this work I have known many aspects of a spectacular person who can teach me so many things...” (AEA).

**Figure 2:**
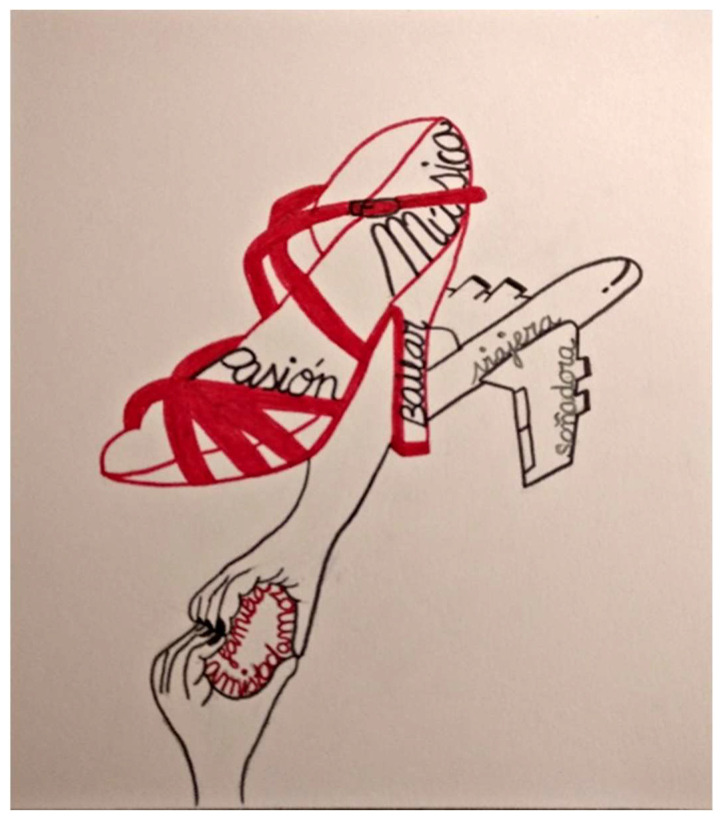
Dreaming. Drawing (CRIGO).

We understand that the arts can generate an improvement in social relationships and self-knowledge from learning by doing, learning by cooperating, and learning from the perspective of affection and sensitivity. Students projected their identities in the life stories and artistic productions, working in reference to their own self and their classmate. This identity contribution is very important because it intensifies the living experience and empowers people to make decisions and learn how to do that. Authors such as Molina et al. [26] highlight how artistic activities encourage socialization, integration and cooperation processes as well as facilitate relationships with people. Moreno [27] also affirm that the arts provide numerous personal benefits such as communication, cultural knowledge, creativity, expressiveness, self-esteem, self-knowledge and self-esteem, self-knowledge, or knowledge of others. Some of the results are shown as an example as in Figure 2.

#### 3.2.2. The Wish to Share Experiences

The wish and motivation to share views and experiences is fundamental for learning, because, in the end, we remember what moves us and what we do. Students should therefore go through the educational process and consider their emotions and feelings.

In the life stories and artistic productions, numerous situations were recorded, reflecting the relevance for students to share experiences with their classmates. “If I stop and think about the long road we have travelled together, I can’t say who taught whom more”. (PAES).

These stories are like a reencounter with the otherness, situating us on the affective and social plane. They show the recognition of the other, of the listener and the speaker. In the case of PAES, she describes a shared vital teaching process, where human nature and emotions intermingle with the academic program. The story conveys her gratitude and admiration thanks to the encounter with her classmate. This is an example where the student manages to express the wish to understand the other person, and to accept her distinctiveness.

#### 3.2.3. Difficulty Expressing Feelings and Emotions

Our records in the field diary show how all the students, at first, had difficulty interacting, and expressing and communicating their emotions. However, during the activities, they gradually acquired the self-confidence to share their emotions with their classmates. 

Considering the first proposal of performative action, “It’s me with another”, the students reacted with a mixture of surprise and concern as this was an unusual activity in the university environment. In addition, they were forced to interact, trying to understand the others and themselves, acquiring certain responsibility to guide their own learning process.

This is included in the field journal, as shown below: “...This is included in the field diary, as follows: “... the students were somewhat expectant They were not sure what to do in the activity. First, they were somewhat shy about taking off their shoes, having to talk with their classmates, and putting on someone else´s shoes. Some students had a hard time deciding which shoes to wear” (DC1S)

This difficulty of expressing and sharing emotions is also observed in the second activity. The conversations between the pairs of students were short, and with brief explanations about their experiences. The topics of these conversations focused on empathy and antipathy experiences in partner and family relationships. Some words and phrases recorded allowed us to assess their degree of empathy: “listening, giving advice to a classmate” (MACA), “helping a friend” (JADO), “moral support for a friend due to the complicated situation of her brother” (ALDO), “because of the death of a friend´s grandfather” (LUI), “Accident of a family member of a childhood friend” (ALMA). 

However, in 40% of the cases, antipathy is expressed. “Relationship of a friend with someone who did not suit her” (JADO), “I met a girl who never agreed with me” (ANPE), “I hardly knew her and criticized everything that I did” (ELVI), “His friends boyfriend cheated on her, and he was glad because he was not a good guy” (MACA), “The death of a dog” (INCRU). 

In the following activities of the project, an improvement of the communication and expression between students was observed. The life stories and artistic creations facilitated the development of personal expression and the recreation of emotional experiences. 

#### 3.2.4. Creation of Alternative Meeting Spaces

During the process, spaces of affective and cognitive meeting were created among the participants. The performative, narrative and visual practice enhanced the experiential exchange for the shared construction of experiences and the creation of new meanings. In particular, the artistic creations and the stories allowed a greater space of affective expression and knowledge. 

The artistic creations brought new experience meanings, turning words into images, or creating new possibilities for expression and creativity. Each visual work was an example of creative and pedagogical exploration linked to the construction of other discourses in educational practices focused on the human nature.

A student said about the project: “... if I had to sum up in a paragraph all that I want to convey with this shoe, this would be it: In life you neither win nor lose, neither fair nor triumph. In life you learn, you grow, you discover. It is written, deleted, and rewritten again. It is spun, frayed, and re-spun, like a shoe when you must tie it again, so fight for your dreams” (ANPE)

The productions also allowed us to think about empathy and experience what the other person feels. The encounter with the other led to a shared affinity embodied in their identities. Some identities were represented in the story and in the visual form as an alternative space. This is possible, because when communicating their experience, both students are involved in a process of reconstruction of oneself, in a personal search for meaning, and talking about the other as a being in relationship.

**Figure 3:**
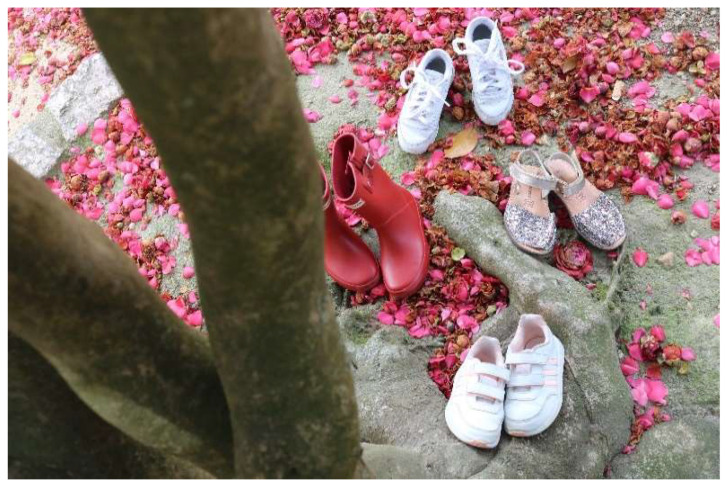
The Pass of time. Photography (PAES).

You can observe this idea in PAESs photograph (Figure 3) representing life, strength and personal achievements through a tree, a learning process that is shared, as she noted: “I have taught her many things, but she may have taught me even more”.

### 3.3. Creativity

The students showed a clear tendency toward high creativity. We observed that the greatest levels occurred in the last activity of artistic creation. This was due to several aspects. On the one hand, as the project unfolded, students felt increasingly more uninhibited and motivated, creating an atmosphere of trust that gave rise to a greater participation and a more creative environment. In our opinion, for the development of creativity, a favorable environment and a positive attitude toward the activities are needed. That is why, one of our findings was that motivation enhanced creativity. This is reflected in a fragment of a life story: “I have done this project with my classmate M, whom I thank for the time, patience, and affection. At the same time, I appreciate the opportunity to participate in this project, because it has helped us to get to know each other better, consolidating and strengthening our friendship bonds” (DAAL)

The activities chosen for our project were open proposals that promoted the development of multisensory creativity, in such a way as to lead to an increase in student motivation and involvement. This was an important aspect since students could experiment and explore with freedom and autonomy the creative process from the perspective of different artistic and sensorial languages.

#### 3.3.1. Originality

Originality was evaluated as a characteristic of creative thinking, such as the production of infrequent and ingenious responses through oral expression, writing or visual production.

**Figure 4:**
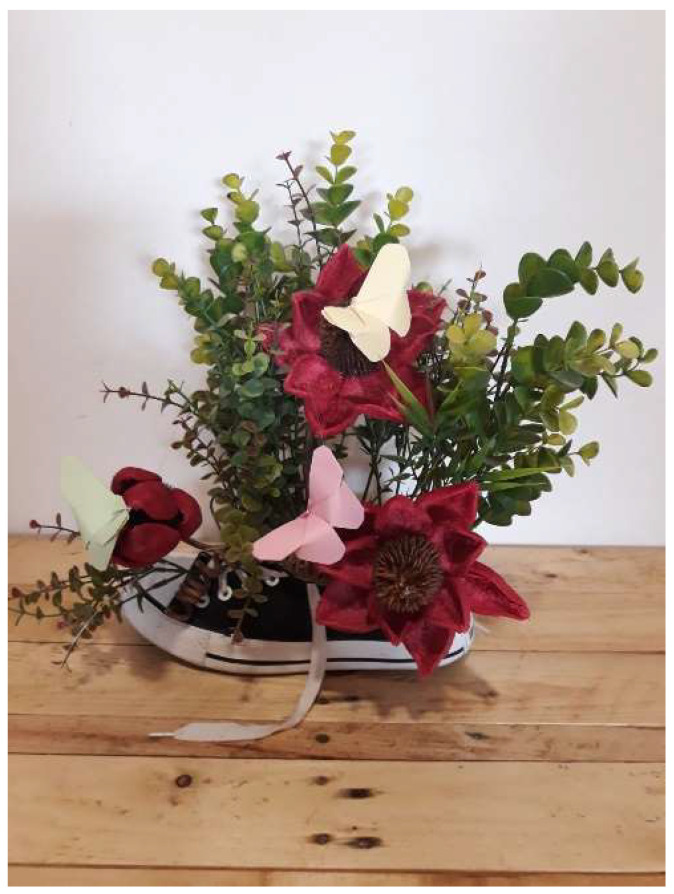
Spring. Photography (INCRU).

The artistic productions were original works, created from their own visual perspective and with the concern for aesthetic and visual quality. As an example of this creative feature, we analyzed the work entitled Spring (Figure 4) where the author inquiries about the possibilities of the still life topic to represent her classmate. The author said that: “If I had to identify my classmate with something, that would be spring. Spring is a period in which plants grow and bloom. The days are sunnier and longer” (INCRU). This work explores the frontier of fiction to interpret a personal story “... It´s freedom. It´s like waking up after winter. Nature that emerges from shoes, that seeks sunlight to grow and open to life... That´s how I see it” (INCRU). Finally, we should note that the image was conceived as a reflective process that challenged us to look for new perspectives within ourselves.

#### 3.3.2. Breaking Boundaries

The breaking of boundaries was understood as the rejection of accepted conceptions or solutions, problematizing what is culturally given. 

In the activities developed, we discovered a degree of reflection which contrasts with automatisms or routine procedures. In the field of self-knowledge and mutual recognition, we consider that students have come to perceive others from different perspectives, which contributes to enhancing their understanding. This is a creative exercise of breaking boundaries aimed at looking differently at what is usually overlooked. “With the second interview, which was much more dynamic, I have been able to explore her way of thinking or see how she feels about certain present-day worldwide problems” (SABE).

In the stories, students gave much importance to be true to themselves, to have their own criteria, to fight for change as a personal quality, and a way of life to be happy. They relate the creative capacity to the improvement of the quality of life and to a way of “overcoming frustration or impotence...” (ITDO).

#### 3.3.3. Composition Esthetics

Esthetics is a form of knowledge related to the Platonic classical sense of creation, sensitive production, and revelation. We assessed the composition esthetics linked to the creation of the self from the perspective of experimenting different ways of composing an idea with an attractive and suggestive visual representation. 

The value of this esthetic, compositional component lies in the sensitive and intellectual knowledge generated to represent the other person through a plastic and visual language.

An example is the photographic series taken by DAAL, whose form of representation reveals a high degree of visual literacy because he can relate the esthetic, conceptual and formal significance in a holistic way. DAAL provides creative continuity to the life story, daring to give a cinematographic look to the composition, by integrating the shoes in a game of lights, colors, planes, and compositional forms. 

**Figure 5:**
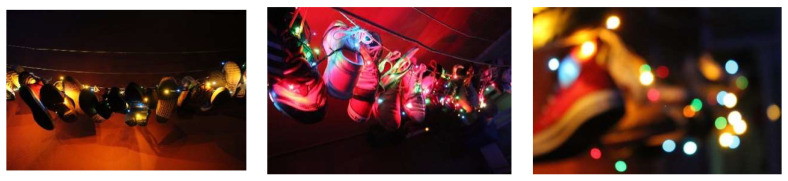
Evocation of Light. Photographic series (DAAL).

The visual discourse generates new knowledge through a sensitive and polysemic result. Photographs can be by themselves an artistic result, and therefore generate knowledge through their esthetic qualities as can bee seen in Figure 5.

## 4. Discussion and Conclusions

The arts have long been promoted as a therapeutic resource [28]. However, there is scarce research on how the arts help people learn to care about others other than themselves.

In fact, art can reconquer the ability to build individual and group identities and subjectivities [29]. Therefore, we attempted to establish in this experience relational processes that forced students to think and act for achieving a more fluid and dynamic cooperation and relationship. In short, the project made it possible to get closer to the other person, with the responsibility of understanding ways of seeing and being in the world, of expressing emotions, sensations or identifying situations through a shared artistic experience.

In this experience, artistic education was conceptualized not only as a plastic activity, but as an educational action where students could think, analyze, photograph, construct, write, listen, draw or walk-through esthetic inquiry. One should keep in mind that positive emotions facilitate memory and learning [30]. 

Multisensory creativity led to an increase in students’ motivation and involvement. Creativity and imagination are important methodological principles when using active methodologies [31]. In addition, neuroscience showed that artistic activities [32,33] encouraged creativity and imagination, involved different brain regions, and promoted the development of cognitive processes [34].

From the documentary analysis it was also deduced that the research in arts can create meeting spaces among the students to develop an empathic pedagogical capacity. The educational action can only exercise its power if we are able to accept the reality of the experiences and that of the other person. In this sense, the activities developed were used to expand artistic knowledge towards a social and relational dimension, with the aim of understanding art as part of life.

The main results point out that when art is used for the construction of an empathic identity, the participants in these creative dynamics find it difficult to express their feelings and emotions, but these processes favor social relationships and mutual recognition, the creation of alternative meeting spaces, and the promotion of creativity. The study presented has limitations. However, this study contributes to become an opportunity to identify new gaps in this field of knowledge and, therefore, to establish new lines of research. As Rusu [35] pointed out, only to the extent that we can be enriched by empathic experiences, we can truly understand those around us. In fact, there is already previous evidence that different artistic manifestations can favor empathy or positive attitudes towards others, thus producing more cooperative relationships [36,37,38].

The current study presents limitations such as the lack of previous research studies on the topic that would allow us to lay solid foundations for understanding the problem under study. However, it is also true that this lack of scientific evidence can become an opportunity to break new ground in this field of knowledge and, therefore, to establish new lines of research.

There have been attempts to triangulate the techniques and not only students’ views were recorded in a field journal, but also those of the teaching staff. However, quantitative instruments could prove useful for profiling in more detail the consequences of the program, such as the use of a satisfaction scale or a scale that measures the different creativity factors.

It would also be interesting to check the effects at a longitudinal level, that is, to check the stability over time of these results.

Finally, we have not considered the cultural and educational limitations of the participants, although it is true that the group is very homogeneous. Normally this bias is negative, but in this study, we consider it a positive bias since all the students participated and achieved practically the same goals.

Artistic quality in the IBA does not respond to an elitist criterion, but rather deprives its communicative value. In this sense, the reactions provoked by the artistic manifestation are more important than the artistic quality as it would be considered from external aesthetic criteria.

More important than the artistic quality as it would be considered from external aesthetic criteria. In this sense, the criterion of communication and social responsibility predominates over that of artistic ability [39]

Based on these results, we suggest some lines of action that will allow the use of art as a means of developing the capacity for empathy and lead to the increase of motivation in the classroom:

To promote among the students a pedagogical reflection on the use of artistic inquiry to favor the exploration and communication of multisensory experiences and a meaningful learning process.

To develop an artistic education for a better understanding of oneself and the recognition of the other, giving the opportunity to exchange and share inferences to generate shared knowledge. 

To create alternative meeting spaces that give the opportunity to experience new ways of looking, listening, feeling through arts.

To develop activities that promote multisensory creativity, helping students to face the hegemony of the unique thought; that is, to have the ability to generate new points of view, respect difference, argue with freedom and critical reflection.

Finally, the composition esthetics should be considered as an important value for the development of creativity because it provides sensitive knowledge, allowing new worldviews and new meanings for our own life.

Therefore, this experience could be useful for university professors who teach subjects related to art education and whose goal is to train students, not only in art education curricular contents but also in transversal competencies (adaptability, communication, social skills, creativity, collaborative work, etc.) and active methodologies. However, it can also be used in the continuing education of teachers at any educational level or for the training of educators in the non-formal field since it can be easily adapted to any educational context and can even be used with other artistic techniques.

## Figures and Tables

**Table 1:** Definition of the categories and indicators thereof.

Categories	Indicators
Esthetic inquiry	New artistic expressions, harmony, emotions, and experiences
Empathic pedagogical relationship	Mutual recognition and self-knowledge
	Wish to share experiences
	Difficulty expressing feelings and emotions
	Creation of alternative meeting spaces
Creativity	Originality
	Breaking boundaries
	Composition esthetics

## Data Availability

The data are not publicly available due to confidentiality reasons.

## References

[1] Fullan M. (2002). Las fuerzas del Cambio. Explotando las Profundidades de la Reforma Educativa [The Forces of Change. Exploiting the Depths of Educational Reform].

[2] Bárcena F., Mélich J.C. (2000). La Educación como Acontecimiento Ético [Education as an Ethical Event].

[3] Hernández F. (2011). Pensar la Relación Pedagógica en la Universidad Desde el Encuentro Entre Sujetos, Deseos y Saberes [Think about the Pedagogical Relationship in the University from the Encounter between Subjects, Desires and Knowledge].

[4] Irwin R.L. (2013). La práctica de la a/r/Tografía [The practice of a/r/tography]. Rev. Educ. Pedagog..

[5] Bourriand N. (2006). Estética Relacional [Relational Aesthetics].

[6] Efland A.D., Freedman K., Stuhr P. (2003). La educación en el Arte Posmoderno.

[7] Walia C. (2019). A dynamic definition of creativity. Creat. Res. J..

[8] Glăveanu V.P. (2013). Rewriting the language of creativity: The Five A’s framework. Rev. Gen. Psychol..

[9] Bridges D., Schendan H.E. (2019). Sensitive individuals are more creative. Personal. Individ. Differ..

[10] Richardson D.C., Street C.N., Tan J.Y., Kirkham N.Z., Hoover M.A., Ghane Cavanaugh A. (2012). Joint perception: Gaze and social context. Front. Hum. Neurosci..

[11] Moya-Albiol L., Herrero N., Bernal M.C. (2010). Bases neuronales de la empatía. Rev. Neurol..

[12] Mestre-Escrivá V., Frías-Navarro M.D., Samper-García P. (2004). La medida de la empatía: Análisis del Interpersonal Reactivity Index. Psicothema.

[13] Amabile T.M., Pillemer J. (2012). Perspectives on the social psychology of creativity. J. Creat. Behav..

[14] Corazza G.E. (2016). Potential originality and effectiveness: The dynamic definition of creativity. Creat. Res. J..

[15] Kou X., Konrath S., Goldstein T.R. (2020). The relationship among different types of arts engagement, empathy, and prosocial behavior. Psychol. Aesthet. Creat. Arts.

[16] Kim K.W., Yang H., Kang S.J. (2019). The Effect on Manifesting Group Creativity by Empathy Level of Students in the Elementary Science Class. J. Korean Elem. Sci. Educ..

[17] Stamatopoulou D. (2018). Empathy and the aesthetic: Why does art still move us?. Cogn. Process..

[18] Lubicz-Nawrocka T. (2019). Creativity and collaboration: An exploration of empathy, inclusion, and resilience in co-creation of the curriculum. Stud. Engagem. High. Educ. J..

[19] Thurber E.F., Eisner E., Day M. (2004). Teacher Education as a Field of Study in Art Education: A Comprehensive Overview of Methodology and Methods Used in Research about Art Teacher Education. Handbook of Research and Policy in Art Education.

[20] Marín-Viadel R. (2011). Las investigaciones en educación artística y las metodologías artísticas de investigación en educación: Temas, tendencias y miradas. Educação.

[21] Martínez Cano S. (2018). Aprender pensando: Metodologías artísticas para la escuela. Padres Maest. J. Parents Teach..

[22] Stake R.E. (2005). Investigación con Estudio de Casos [Case Study Research].

[23] Yacuzzi E. (2005). El Estudio de Caso como Metodología de Investigación: Teoría, Mecanismos Causales, Validación [The Case Study as a Research Methodology: Theory, Causal Mechanisms, Validation]. EconPapers.

[24] Marín Viadel R., Roldán J. (2017). Ideas Visuales. Investigación Basada en Artes e Investigación Artística.

[25] Knight L., Cutcher A.L. (2018). Arts-Research-Education. Connections and Directions.

[26] Molina M.C., Pastor C.I., Violant V. (2009). Guia D’estratègies Ludicocreatives per al Voluntariat de Ciber Caixa Hospitalària.

[27] Moreno A. (2010). La mediación artística: Un modelo de educación artística para laintervención social a través del arte. Rev. Iberoam. Educ..

[28] Creswell J.W. (2009). Research Design: Qualitative, Quantitative, and Mixed Methods Approaches.

[29] Fernández F. (2002). El análisis de contenido como ayuda metodológica para la investigación [Content analysis as a methodological aid for research]. Cienc. Soc..

[30] Neuendorf K.A. (2002). The Content Analisis Guidebook.

[31] Fancourt D., Finn S. (2019). What is the Evidence on the Role of the Arts in Improving Health and Well-Being?.

[32] Casafont R. (2014). Viaje a tu Cerebro Emocional [Travel to Your Emotional Brain].

[33] Hardiman M., Rinne L., Yarmolinskaya J. (2014). The effects of arts integration on long-term retention of academic content. Mind Brain Educ..

[34] Wandell B., Dougherty R.F., Ben-Shachar M., Deutsch G.K., Asbury C., Rich C. (2008). Training in the arts, reading, and brain Imaging. Learning, Arts, and the Brain.

[35] Rusu M. (2017). Empathy and communication through Art. Rev. Artist. Educ..

[36] Bang A.H. (2016). The restorative and transformative power of the arts in conflict resolution. J. Transform Educ..

[37] Rieger K., Chernomas W., McMillan D., Morin F., Demczuk L. (2016). Effectiveness and experience of arts-based pedagogy among undergraduate nursing students: A mixed methods systematic review. JBI Database Syst. Rev. Implement Rep..

[38] Campos L., Dias P., Duarte A., Veiga E., Dias C.C., Palha F. (2018). Is it possible to “find space for mental health” in young people? Effectiveness of a school-based mental health literacy promotion program. Int. J. Environ. Res. Public Health.

[39] Finley S. (2003). Arts based inquiry in QI: Seven years from crises to guerrilla warfare. Qual. Inq..

